# PP2A-Twins Is Antagonized by Greatwall and Collaborates with Polo for Cell Cycle Progression and Centrosome Attachment to Nuclei in Drosophila Embryos

**DOI:** 10.1371/journal.pgen.1002227

**Published:** 2011-08-11

**Authors:** Peng Wang, Xavier Pinson, Vincent Archambault

**Affiliations:** Institut de Recherche en Immunologie et en Cancérologie and Département de Biochimie, Université de Montréal, Montréal, Canada; Stowers Institute for Medical Research, United States of America

## Abstract

Cell division and development are regulated by networks of kinases and phosphatases. In early Drosophila embryogenesis, 13 rapid nuclear divisions take place in a syncytium, requiring fine coordination between cell cycle regulators. The Polo kinase is a conserved, crucial regulator of M-phase. We have recently reported an antagonism between Polo and Greatwall (Gwl), another mitotic kinase, in Drosophila embryos. However, the nature of the pathways linking them remained elusive. We have conducted a comprehensive screen for additional genes functioning with *polo* and *gwl*. We uncovered a strong interdependence between Polo and Protein Phosphatase 2A (PP2A) with its B-type subunit Twins (Tws). Reducing the maternal contribution of Polo and PP2A-Tws together is embryonic lethal. We found that Polo and PP2A-Tws collaborate to ensure centrosome attachment to nuclei. While a reduction in Polo activity leads to centrosome detachments observable mostly around prophase, a reduction in PP2A-Tws activity leads to centrosome detachments at mitotic exit, and a reduction in both Polo and PP2A-Tws enhances the frequency of detachments at all stages. Moreover, we show that Gwl antagonizes PP2A-Tws function in both meiosis and mitosis. Our study highlights how proper coordination of mitotic entry and exit is required during embryonic cell cycles and defines important roles for Polo and the Gwl-PP2A-Tws pathway in this process.

## Author Summary

The development and survival of all living organisms relies on the fine regulation of cell division at the molecular level. This coordination depends on kinases and phosphatases, enzymes that catalyze the addition and removal of phosphate groups on specific target proteins. The genes encoding these enzymes have been largely conserved between species during evolution. In a previous paper published in *PLoS Genetics*, we found an antagonism between the Polo and Greatwall mitotic kinases in the fruit fly model. In this study, we have used fly genetics to identify additional genes that function with *polo* and *greatwall* during early embryogenesis. We have found a specific form of the Protein Phosphatase 2A (PP2A-Tws) that collaborates with the Polo kinase at a stage when multiple nuclei rapidly divide in a large, single-cell early embryo. We found that Polo and PP2A-Tws are both required for the proper cohesion between nuclei and the centrosomes, which are essential structures for mitosis and embryonic development. We also found that the Greatwall kinase antagonizes the PP2A-Tws phosphatase to promote mitosis and meiosis. Our genetic study sheds new light on cell cycle regulation and is consistent with recent results from biochemical studies using frog cell extracts.

## Introduction

The cell cycle is largely driven by networks of kinases and phosphatases that coordinate the sequential events of cell division in addition to regulating each other [1]. Kinases of the Polo, Aurora and cyclin-dependent families play particularly important roles in this process [2]–[4]. Phosphatases compete with kinases for the same substrates, and the balance between their activities is subjected to a fine regulation through the cell cycle [5], [6]. While in budding yeast the Cdc14 phosphatase plays a crucial role in promoting mitotic exit by dephosphorylating Cdk1 substrates and promoting its inactivation [7], it is becoming increasingly clear that a form of Protein Phosphatase 2A (PP2A) bound to a B-subtype adaptor subunit fulfills this function in vertebrates [8], [9]. Yet, a clear picture of the dynamic interplay between specific kinases and phosphatases during the cell cycle is still missing.

The developmental program of a complex organism requires that the cell cycle machinery adapt to the situation and contribute to the integration of cell divisions in various tissue contexts and cell types. Early embryogenesis typically involves rapid cell cycles where S-phases and M-phases alternate rapidly with little or no gap phases, growth or transcription. In Drosophila, the first 13 mitotic cycles occur in a syncytium at around 10–15 min intervals with virtually no zygotic transcription, and are driven by maternally contributed proteins and mRNAs [10]. At that stage, nuclei migrate in the syncytium, first in an axial fashion, and then towards the cortex to form the blastoderm [11]. In addition to organizing mitotic spindles, centrosomes are tethered to nuclei in the syncytium and constitute anchors for the nuclei to a network of anti-parallel astral microtubules (MTs) that push nuclei away from each other and towards the cortex [11]. Because of the absence of G1 at that stage, DNA replication and centrosome duplication occur shortly after mitotic exit and as early as telophase [10]. The nuclear envelope does not completely break down during syncytial mitoses, but becomes fenestrated to allow MTs to penetrate nuclei [12]–[14]. While centrosomes are dispensable for cell division in many cell types, they are absolutely essential for early embryogenesis in Drosophila [15], [16].

The Polo kinase is a conserved, central regulator of M-phase [2], [17], [18]. Polo promotes mitotic entry by activating Cdc25 phosphatases that activate Cdk1/Cdc2 [19], [20]. Cyclin B-Cdk1 triggers nuclear envelope breakdown and chromosome condensation [21], [22]. Polo also plays important roles in centrosome maturation, chromosome attachment to MTs, bipolar spindle assembly and cytokinesis [2], [18]. However, how Polo functions contribute to various developmental contexts has been little explored. We have recently identified the Greatwall kinase genetically in an antagonistic functional relationship with Polo in the Drosophila syncytial embryo [23]. Decreasing Polo activity and increasing Gwl activity together lead to a failure in early embryogenesis characterized by centrosome detachments from nuclei. Discovered in Drosophila as an important mitotic kinase [23], [24], Greatwall has rapidly emerged as a crucial regulator of M phase in Xenopus extracts [25] and in human cells [26], [27]. The precise nature of the functional relationship between Polo and Greatwall that we uncovered genetically remained elusive [23].

Free centrosomes can occur in several distinct ways in syncytial embryos, detaching from mitotic spindles or interphase nuclei, or duplicating independently from nuclei [28]. In response to DNA damage, centrosomes can be inactivated, leading to the loss of the damaged nucleus which sinks into the yolk [29]. This response depends on the Chk2 kinase [30]. Mutations inactivating *chk2* did not prevent the centrosome detachments observed in embryos where Polo function was decreased and Gwl function was increased [23], suggesting that those events are not due to an activation of the DNA damage checkpoint, but reflect problems in coordinating the early mitotic divisions at another level.

We have conducted a genetic screen to identify additional genes functioning with *polo* and *gwl*. We uncovered two genes encoding subunits of PP2A as the strongest hits in this screen: the catalytic subunit gene (*microtubule star/mts*) and the B-type adaptor subunit gene (*twins/tws*). Phenotypic examination of the nuclear divisions in single mutants shows that Polo promotes the cohesion between centrosomes and nuclei around prophase while PP2A-Tws promotes centrosome attachment to nuclei during mitotic exit. Compromising Polo and PP2A-Tws functions simultaneously strongly enhances centrosome detachments and leads to failures in syncytial embryonic development. Moreover, we show that the Gwl kinase functions to antagonize PP2A-Tws in both meiosis and mitosis. Our results indicate that precise coordination of mitotic entry and exit is crucial during embryonic cell cycles, and implicate Polo, Gwl and PP2A-Tws as key regulators. We discuss our findings in the context of recent studies from the Xenopus extract and Drosophila systems, which also implicate a pathway linking Gwl and PP2A in regulating M phase.

## Results

### Polo function is required for proper cohesion between centrosomes and nuclei

**Figure 1:**
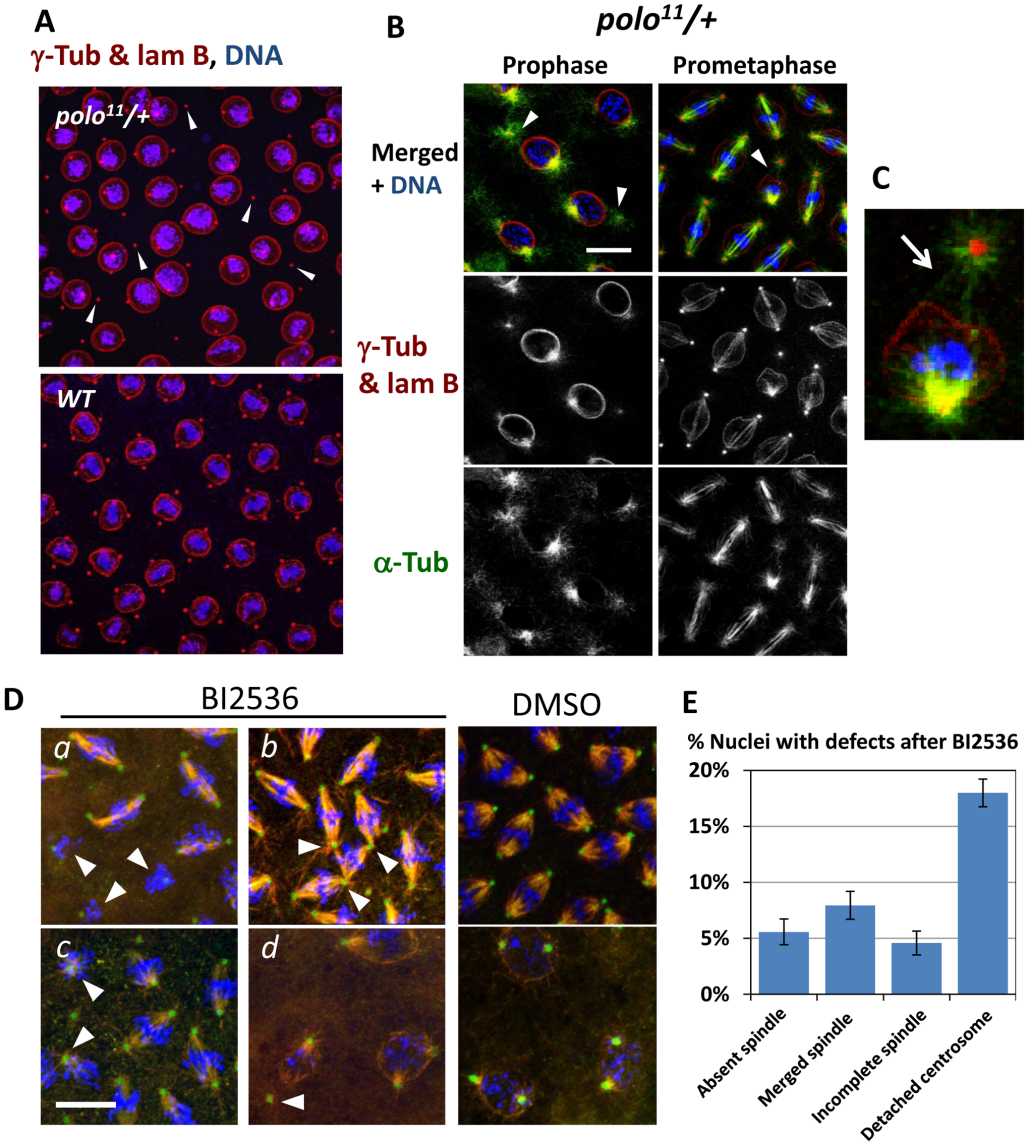
The Polo kinase is required for proper centrosome attachment to nuclei in syncytial embryos. A, B. Centrosome detachments observed in prophase in embryos from *polo^11^/+* mothers (arrowheads). B. Embryos from *polo^11^/+* mothers. Left: detachments in prophase; Right: example of a detachment that persists in prometaphase. C. Enlargement from the prometaphase image in B. Arrow: MTs from the detached centrosome fail to penetrate the nuclear envelope. D. Chemical inhibition of Polo results in centrosome detachments and spindle defects consistent with known Polo functions: *a*: absent spindles; *b*: fused spindles; *c*: incomplete spindles; *d*: detached centrosomes. Embryos were treated with 1 µM BI2536 for 30 min to inhibit Polo. Red: α-Tubulin; Green: γ-Tubulin, Blue: DNA. **E**. Quantification of the observed defects. N = 19 embryos; Error bars: S.E.M. The frequency of all scored defects in control embryos (DMSO only) was less than 1% (N = 8). Scale bars: 10 µm.

Halving the levels of Polo kinase in the fly does not cause any obvious problems of development or fertility. However, embryos laid by *polo* heterozygous females are made inviable by a gain of Greatwall (Gwl) function or by overexpression of Map205, a strong physical interactor of Polo [23], [31]. In both cases, defective embryos are characterized by a strikingly penetrant phenotype of centrosome detachments from nuclei. These observations prompted us to examine more closely the effects of compromising Polo function in the syncytial embryo. Immunofluorescence in embryos from mothers heterozygous for the null *polo^11^* allele [23] shows that several nuclei in prophase have one dislocated centrosome (Figure 1A, 1B left, arrowheads, quantified in Figure 5B), consistent with previous results [23]. This phenotype is also observed in embryos from mothers heterozygous for *polo^9^*, a strong hypomorph (data not shown). Centrosome detachment can also be observed in prometaphase/metaphase (Figure 1B right). Thus, the cohesion between centrosomes and nuclei in early mitosis is sensitive to Polo kinase levels.

The Polo kinase is essential for a wide variety of functions during cell division, including centrosome maturation, spindle assembly and cytokinesis. Yet, these processes do not appear compromised in embryos receiving half their normal amount of Polo, while centrosome attachment to nuclei is partially defective. This suggested that centrosome-nuclei cohesion in the embryo is particularly sensitive to a decrease in Polo activity, while the other functions of Polo are satisfied with a lower Polo level.

We sought to examine the effects of a more severe reduction in Polo function during syncytial embryogenesis. Because Polo is contributed to the embryo maternally, and because it is essential for viability and for meiosis, we could not examine the effects of a complete genetic inactivation of Polo on embryogenesis. To circumvent these limitations, we used chemical inhibition. We found that the Polo-like kinase 1 inhibitor BI2536 [32] is a potent inhibitor of Drosophila Polo. Treatment of D-Mel or S2 cells in culture with BI2536 phenocopied RNAi depletion of Polo, leading to a higher mitotic index and an accumulation of prometaphase cells that often displayed monopolar spindles (data not shown). As predicted, treatment of embryos with BI2536 led to a high frequency of centrosome detachment, along with other defects typical of Polo inhibition in cultured cells, including incomplete or absent spindles and misaligned chromosomes (Figure 1D, 1E). Not surprisingly, defective, neighbouring spindles were often fused in the syncytium (Figure 1D*b*, 1E). However, spindle defects were less penetrant than centrosome detachments (Figure 1E). All defects were almost never observed (<1%) in control embryos treated with DMSO alone. Altogether, our results suggest that a lower level of Polo activity is sufficient for spindle assembly and function, while proper cohesion between centrosomes and nuclei requires a higher level of Polo activity.

### Transiently detached centrosomes are recaptured by mitotic spindles

**Figure 2:**
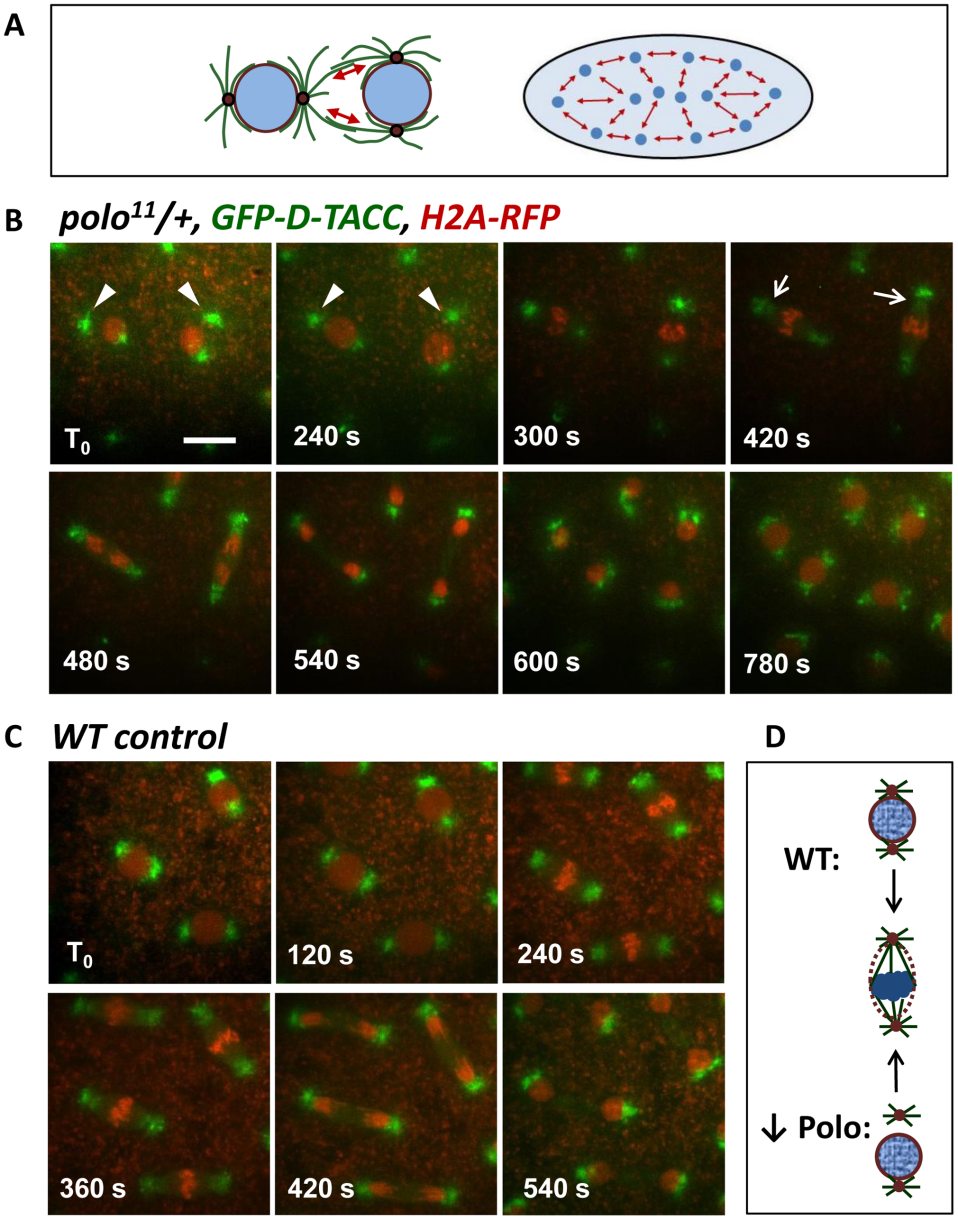
Detached centrosomes from nuclei can be recaptured by the mitotic spindles. A. During syncytial divisions, nuclei are linked by anti-parallel astral MTs (left), which push nuclei apart (red arrows) and towards the cortex (right). B. Time-lapse imaging of embryos from *polo^11^/+* mothers and expressing GFP-D-TACC to mark centrosomes and spindles and H2A-RFP to mark the chromatin. At T_0_, detached centrosomes (arrowheads) are clearly away from the nuclei. At 420 s, detached centrosomes are recaptured (arrows) and nuclear division is completed normally. Scale bar: 10 µm. C. Time-lapse imaging of a *WT* embryo as a control. D. Schematic illustration of a nuclear division in an embryo with reduced Polo levels, vs a normal, WT embryo. Centrosome detachment in the Polo-compromised embryo is only transient.

Centrosome-nuclei cohesion is crucial to embryonic development, since it provides the link between nuclei and a skeleton of anti-parallel astral microtubules (MTs) that pushes the nuclei apart and towards the cortex (Figure 2A), although overlapping MTs are difficult to observe [11]. Moreover, centrosomes are essential to the assembly of bipolar mitotic spindles in syncytial embryos [15]. Because the examined *polo*-compromised embryos are able to complete development, the dislocation between centrosomes and nuclei observed either leads to problems that occur at a tolerated rate, or is transient and does not usually lead to mitotic or nuclear migration defects. To examine these possibilities, we used time-lapse microscopy, following GFP-D-TACC as a centrosomal and spindle marker [33], and histone H2A-RFP as a nuclear marker. In embryos from *polo^11^/+* mothers, we could readily find several nuclei where one centrosome was dislocated from its nucleus of origin (Figure 2B, arrowheads). Yet, dislocated centrosomes were recaptured during spindle assembly (420 s, arrows), and nuclear divisions could then be completed (see also Video S1). This detachment was not observed in embryos from *WT* mothers (Figure 2C and Video S2). These observations explain how the centrosome dislocations seen in *polo*-compromised embryos are transient and have no lethal consequences on their own (Figure 2D). This centrosome recapture has also been observed in embryos with reduced Polo function and elevated Gwl function, although those embryos were much sicker and inviable, and many centrosomes were not recaptured [23].

That Polo is required to keep the cohesion between centrosomes and nuclei is consistent with its known functions at mitotic entry, in promoting centrosome maturation and Cdk1 activation [2], [20]. Polo also assists Cyclin B-Cdk1 in promoting nuclear envelope breakdown [34], [35]. In Drosophila syncytial embryos, the nuclear envelope does not completely break down in mitosis, but becomes fenestrated near centrosomes, allowing MTs to penetrate nuclei and reach chromosomes [12]–[14]. At that stage, centrosome attachment to the nuclear envelope is weakened and is replaced by MT attachments to chromosomes. Thus, the detached centrosomes observed in prophase when Polo activity is decreased could be explained by a failure to coordinate centrosome function with nuclear envelope fenestration at mitotic entry. Consistent with this idea, in *polo*-compromised embryos, MTs emanating from the detached centrosome can be seen as if pressing on the nuclear envelope, which caves in deeply but does not allow MT penetration at the prophase/prometaphase transition, while MTs from the attached centrosomes have already invaded the nucleus (Figure 1C).

### A genetic screen identifies PP2A-Tws as a strong functional interactor of Polo and Gwl in the syncytial embryo

**Figure 3:**
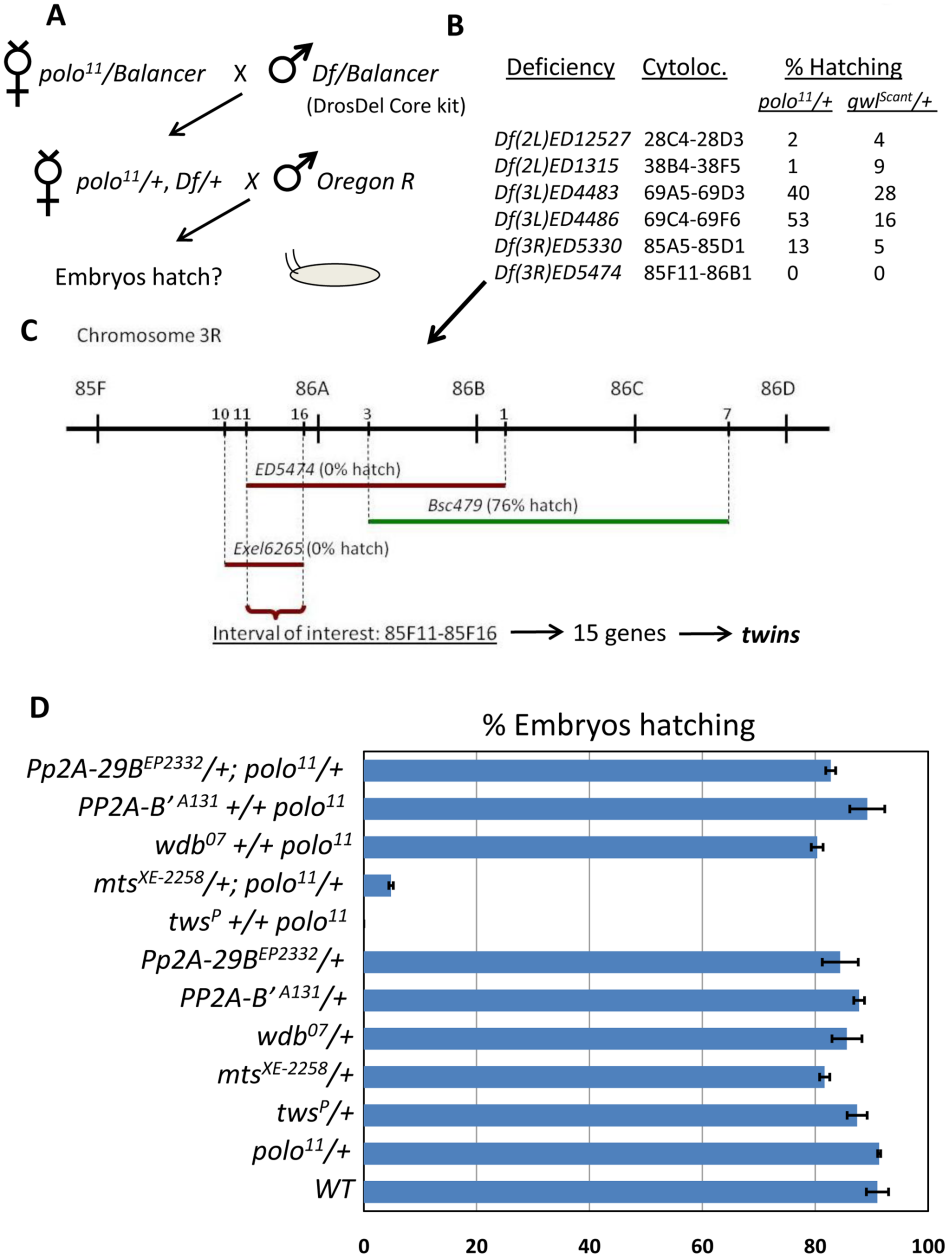
A screen for genes functioning with *polo* identifies the PP2A subunit genes *twins* and *microtubule star*. A. Genetic scheme of the screen. Deficiencies of the DrosDel Core kit were combined with one allele of *polo^11^*. Doubly heterozygous females were taken and their embryos were tested for the ability to hatch. A similar screen was performed with *gwl^Scant^* instead of *polo^11^*. B. Deficiencies obtained that yielded less than 50% embryo hatching in the screen when combined with *polo^11^* or *gwl^Scant^*. For each deficiency, the cytolocation and the percentages of embryos hatching obtained in the screen are indicated. C. Identification of *twins* (*tws*) as a genetic interactor. Testing deficiencies overlapping with *Df(3R)ED5474* for their effect on embryo hatching when introduced in the maternal *polo^11^/+* background allowed to restrict the interval of interest to 15 genes which included *twins*. D. *polo* genetically interacts specifically with *tws* and *mts*. Percentage of embryos hatching for the indicated maternal genotypes. N = 4; error bars: S.E.M.).

The observed centrosome-nucleus cohesion defects in embryos from *polo*-heterozygous females suggested that it could provide a good sensitized background to screen for genes that function with *polo* in the embryo. We conducted such a screen using the DrosDel deficiency core kit, consisting of a sub-collection of large genomic deletions which altogether uncover approximately 60% of the fly genome [36]. Females heterozygous for the *polo^11^* null mutation were systematically crossed to males heterozygous for a single deficiency (Figure 3A). In F1 progeny, females heterozygous for both *polo^11^* and the deficiency were tested for their ability to produce viable embryos, hatching into larvae. The same scheme was applied to test each deficiency in combination with one copy of *gwl^Scant^*, a gain-of-function allele of *gwl* identified previously as a dominant synthetic lethal enhancer of *polo* hypomorphic embryos [23], [37]. The large majority of deficiencies allowed full fertility when combined with *polo^11^* or *gwl^Scant^*. Only 6 deficiencies resulted in less than 50% embryo hatching when combined with either *polo^11^ or gwl^Scant^* (Figure 3B). Interestingly, the deficiencies that interacted with *polo^11^* tended to also interact with *gwl^Scant^* (and vice-versa), further suggesting a very close functional link between Polo and Gwl. We reasoned that the deletions identified were likely to uncover genes that function with *polo* and *gwl*.

Among the deficiencies identified in the screen, the strongest genetic interactor with *polo* and *gwl* was *Df(3R)ED5474*, which resulted in complete failure of the embryos to hatch (Figure 3B). Using overlapping deficiencies, we were able to limit the interval of interest to a region containing only 15 genes on chromosome 3R (Figure 3C). One of those genes was *twins* (*tws*), which encodes a B-type adaptor subunit of PP2A previously implicated in cell cycle regulation [38]-[40] and is the sole ortholog of the human B55 group of adaptor subunits [41]. Combining one copy of *tws^P^* (a strong hypomorphic allele due to a P-element insertion) with one copy of *polo^11^* in the maternal genotype resulted in a complete failure of embryos to hatch (Figure 3D). Similar results were obtained with another allele, *tws^aar-1^*. We did not test if mutations in the other 14 genes uncovered in the interval of interest genetically interact with *polo.* In any case, the genetic interaction between *polo* and *tws* pointed at a functional interdependence between Polo and PP2A in the embryo.

We then tested systematically all PP2A subunit genes in Drosophila for which we could obtain mutants, for potential genetic interactions with *polo* in the same assay. Of the three PP2A adaptor subunit genes tested (*tws, widerborst, PP2A-B'*) only *tws* showed a genetic interaction with *polo^11^* (*CG4733* (*PP2A-B''*) was not tested). Females heterozygous for both *polo^11^* and a mutant allele of *microtubule star* (*mts*), the catalytic subunit gene [42], produced semi-viable embryos (Figure 3D). Similar genetic interactions were observed between *polo^9^*, a strong hypomorph, and *tws* or *mts* (data not shown). Interestingly, *mts* is uncovered by *Df(2L)ED12527*, identified as another strong hit in our screen (Figure 3B). One copy of a mutant allele of *Pp2A-29B*, which encodes the structural subunit of PP2A had no effect in combination with *polo^11^*. This could be explained if this subunit were to be present in excess relative to the other subunits of the holoenzyme. No genetic interactions were detected with deficiencies uncovering *widerborst*, *PP2A-B'* or *Pp2A-29B* (data not shown). Strong genetic interactions were also observed between *gwl^Scant^* and *tws* or *mts* in the same assay. The percentage of embryos hatching from *mts/+*; *gwl^Scant^ /+* and *gwl^Scant^ +/+ tws^P^* was zero in both cases (0; N = 4). These results point at PP2A-Tws as a critical functional interactor of Polo and Gwl during syncytial embryo development. The fact that the two strongest hits out of the 60% of the genome that was screened are subunits of PP2A-Tws highlights the specificity of the pathway uncovered here. We note that a strong genetic interaction was also detected between *Df(2L)ED1315* and *polo^11^* or *gwl^Scant^*; and therefore this deletion could uncover another important gene functioning with Polo, Gwl and PP2A. However, *Df(2L)ED1315* disrupts 94 genes, and the deficiency mapping of the gene of interest is proving challenging.

### Greatwall antagonizes PP2A-Tws in M-phase

**Figure 4:**
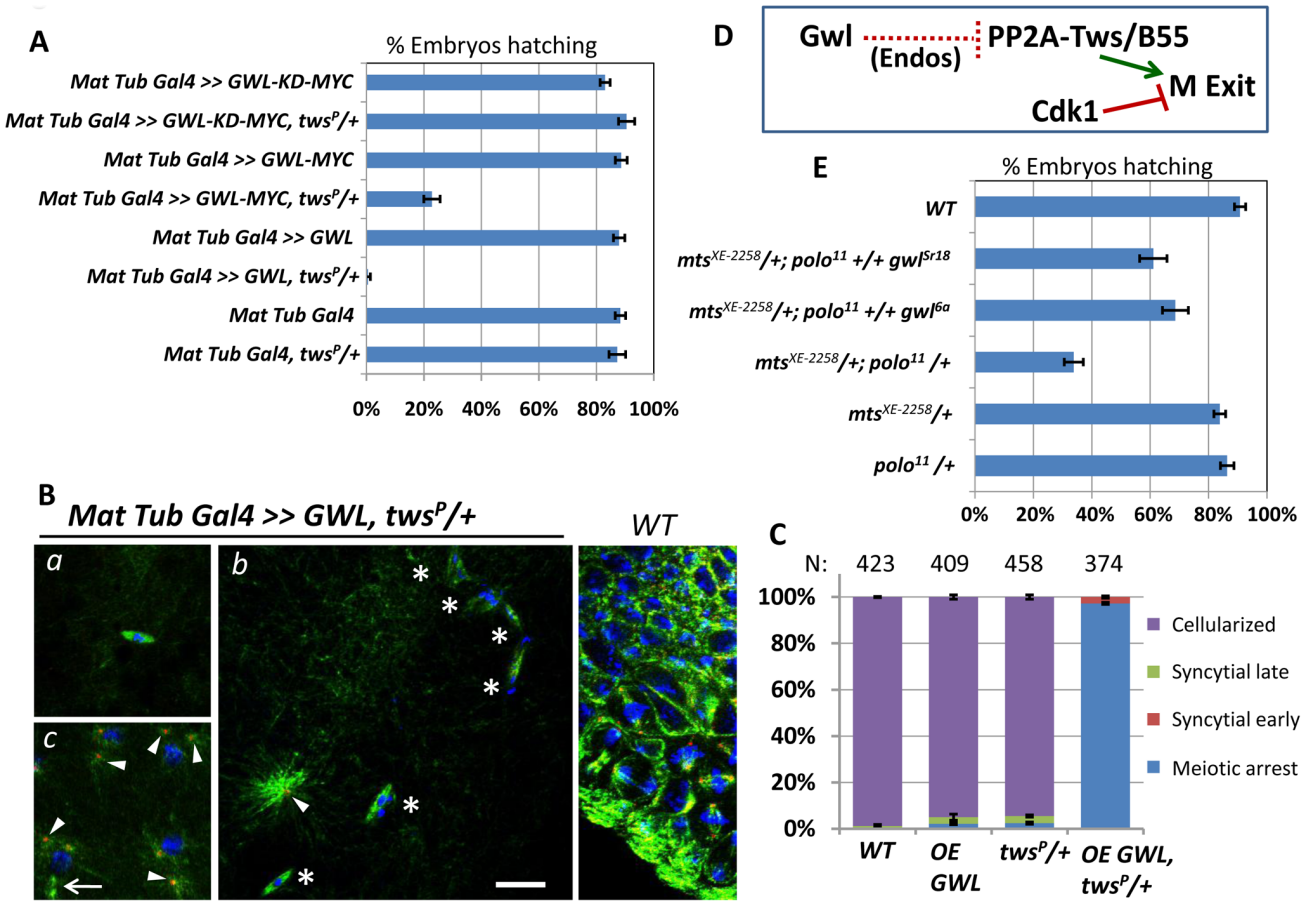
Greatwall antagonizes PP2A-Tws in meiosis and mitosis. A. Overexpression of Gwl in the embryo with reduced dosage of Tws leads to a failure to hatch. This effect is dependent on the kinase activity of Gwl (KD: Kinase-dead). Results shown for the transgenic genotypes combine values obtained for 2 independent transgenic lines. N = 5; Error bars: S.E.M. B. Examples of embryos between 4 to 6 hrs post-laying. *WT* embryos are cellularized by that time (right). *a*: Most embryos overexpressing Gwl and with reduced Tws fail to exit metaphase of meiosis I, where a single acentrosomal meiotic spindle is observed. *b*: Some embryos attempt mitotic cycles and are mostly blocked with multiple aberrant structures, containing condensed chromatin and MTs (asterisks). *c*: mitotic divisions in karyokinesis (rarely seen). Note the presence of a central spindle (arrow). Centrosomes are detached from nuclei (*b, c*; arrowheads). Scale bar: 10 µm. C. Quantitation of the different stages observed for the indicated genotypes in embryos 4 to 6 hours post-laying. *OE GWL*: Overexpression of *UASp-GWL*. The number of eggs/embryos examined is indicated above each column. Error bars: S.E.M. D. Model: Greatwall antagonizes PP2A-Tws to prevent M-phase exit (see text for discussion). E. Halving the amount of Gwl in embryos from mothers heterozygous for both *polo^11^* and *mts^ XE-2258^* mutations partially rescues their ability to hatch. N = 4; Error bars: S.E.M.

The synthetic maternal-effect embryonic lethality between *gwl^Scant^* and *tws* or *mts* heterozygous mutations suggested an antagonistic relationship between Gwl and PP2A-Tws. The *gwl^Scant^* mutant allele of *gwl* leads to a K97M substitution that makes the kinase hyperactive in vitro [23]. To test directly if embryos with reduced PP2A-Tws function are sensitive to a gain in Gwl kinase activity, we overexpressed Gwl in the egg and early embryos. This was achieved by the late female germline expression of Gal4 under control of the maternal α-tubulin promoter, leading to the Gal4-driven expression of *UASp-GWL* in that tissue. Overexpression of Gwl in eggs/embryos from *tws^P^* heterozygous mothers was almost completely lethal, while overexpression of a kinase-dead form of Gwl had no effect on embryonic viability (Figure 4A). Therefore, excessive Gwl kinase activity relative to PP2A-Tws activity disrupts either oogenesis and/or embryonic development.

Fertilised eggs or embryos overexpressing Gwl and with a reduced Tws dosage were examined by immunofluorescence. Strikingly, most eggs/embryos were blocked in metaphase of meiosis I, even after 4 to 6 hours post-laying (Figure 4B*a*, 4C). Embryos overexpressing Gwl alone or with a reduced dose of Tws alone were almost all cellularized by that time, like *WT* embryos (Figure 4B right, 4C). These results suggest a role for Gwl in promoting the meiotic arrest by antagonizing PP2A-Tws. Conversely, we previously showed that a loss of Gwl function in oocytes leads to a failure to arrest in meiosis, associated with unstable sister chromatid cohesion [23]. Our results are consistent with recent biochemical studies showing that Gwl promotes M-phase in Xenopus egg extracts by antagonizing PP2A-B55δ (ortholog of Twins), which has itself been shown to promote M-phase exit by dephosphorylating Cdk1 substrates (Figure 4D, discussed below) [9], [43], [44].

Although most eggs overexpressing Gwl and with a reduced amount of Tws arrested in meiosis, a few eggs managed to complete meiosis and attempted to initiate embryonic development. However, theses embryos usually aborted in the first mitoses, displaying several small aberrant structures with condensed chromatin at the center of small spindles (Figure 4B*b*, 4C). The few mitotic nuclei observed showed a very high incidence of detached centrosomes (Figure 4B*b*,*c*, arrowheads). These observations suggest that the Gwl-PP2A-Tws pathway can also regulate mitotic divisions.

**Figure 5:**
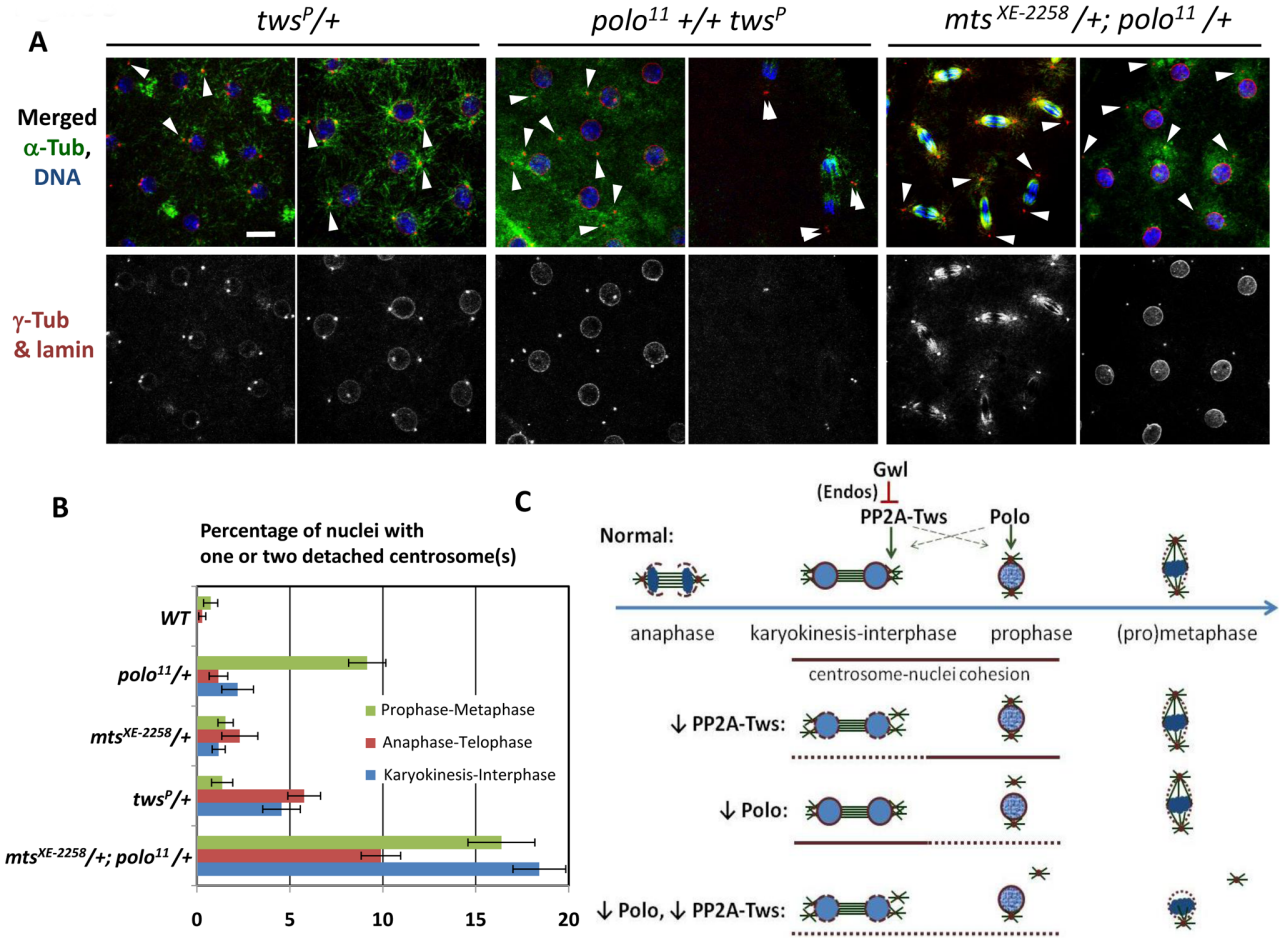
PP2A-Tws collaborates with Polo to promote cell cycle progression and centrosome cohesion to nuclei. A. Images of embryos from mothers of the indicated genotypes. Left: Reduced levels of Tws lead to centrosome detachments in late M-phase that can persist in interphase. Middle and right: reducing both Polo and Mts or Tws strongly enhances centrosome detachments. Scale bar: 10 µm. B. Quantitation of centrosome detachments at different cell cycle stages for the indicated genotypes. Between 5 and 18 embryos were scored for each category. Error bars: S.E.M. C. Model: Polo and PP2A-Tws collaborate to ensure centrosome attachment during the syncytial cell cycles. See text for details.

We reasoned that if normal levels of Gwl regulate mitosis by antagonizing PP2A-Tws, then decreasing the activity of Gwl could rescue the viability of embryos from *mts^ XE-2258^/+; polo^11^/+* mothers, which are semi-viable and show severe mitotic defects (Figure 5A, 5B). Indeed, those embryos were partially rescued by the introduction of one copy of the null allele *gwl^6a^*, or one copy of *gwl^Sr18^*, which abolishes the only maternally contributed splice variant of *gwl* (Figure 4E) [23]. This result strongly suggests that Gwl normally negatively regulates PP2A-Tws during the mitotic divisions in addition to meiosis *in vivo*.

### PP2A-Tws collaborates with Polo to ensure centrosome cohesion to nuclei and nuclear divisions in the syncytial embryo

Phenotypic examination of embryos from *polo*-heterozygous mothers revealed an elevated frequency of transient centrosome dislocations from nuclei in prophase (Figure 1, Figure 5B). Because of the strong genetic interaction between *polo* and *tws* or *mts*, we examined embryos from mothers heterozygous for *tws* and *mts* mutations to reveal any potential defects. Interestingly, we detected a significant incidence of centrosome detachments in both cases. However, unlike those observed in *polo*-compromised embryos which occurred mostly in prophase, centrosome dislocation tended to occur in late M-phase (between anaphase and karyokinesis) in embryos with reduced PP2A-Tws (Figure 5A, 5B).

As expected, embryos combining reductions in Polo and PP2A-Tws levels displayed a strongly elevated incidence of centrosome detachments. Embryos from mothers heterozygous for *polo^11^* and *mts^XE-2258^*, of which a minority were able to hatch into larvae (Figure 3D), showed a high frequency of detached centrosomes at any stage of the mitotic cycles (Figure 5A, 5B). Embryos from mothers heterozygous for *polo^11^* and *tws^P^*, which all failed to hatch (Figure 3D), aborted very early during syncytial divisions, with most or all centrosomes detached already in the first few cycles (Figure 5A). Therefore, Polo and PP2A-Tws collaborate to ensure proper centrosome cohesion to nuclei and cell cycle progression during early embryogenesis. Since the centrosome detachments that occur upon reduction in Polo function are seen mostly in prophase while those that occur when PP2A-Tws is reduced occur mostly in late M-phase, many centrosomes in double mutants may never be able to recover their attachment and drift away, leading to a failure in nuclear division (Figure 5C), as seen in embryos from *polo^11^-Scant* females [23].

## Discussion

In this study, we have explored the functional and molecular relationship between the Polo and Gwl kinases. Our previous study pointed at the importance of proper coordination between these enzymes in the embryonic cell cycles in Drosophila [23]. Our genetic screen has lead to the identification of PP2A-Tws as a potent collaborator with Polo in promoting the cohesion between centrosomes and nuclei. Moreover, our results are consistent with a model where Greatwall antagonizes PP2A-Tws to promote M-phase, and where PP2A-Tws promotes exit from mitosis and meiosis. This model is strongly supported by recent biochemical studies using the Xenopus extract system.

### Regulation of mitosis and centrosome attachment in the rapid embryonic cell cycles

Our results shed new light on cell cycle regulation and syncytial embryogenesis. We clearly show that high Polo activity is needed to promote the normal cohesion between centrosomes and nuclei, and this is mostly observable around the time of mitotic entry. Interestingly, transiently detached centrosomes can be recaptured by the assembling spindle and nuclear division can then be completed. This centrosome recapture is probably essential for successful development of the syncytial embryo. Our systematic genetic screen unveiled a very strong and specific functional link between Polo and a specific form of PP2A associated with its B-type subunit Tws. We also show that PP2A-Tws activity is required for centrosome cohesion with nuclei, although in late M-phase, around the time of mitotic exit. This is consistent with a recent study where centrosome defects were observed in late M-phase when the small T antigen of SV40, which binds PP2A, was expressed in Drosophila embryos [45]. PP2A-B55δ has been recently implicated in promoting mitotic exit in vertebrates, by inactivating Cdc25C and by directly dephosphorylating Cdk1 mitotic substrates [43], [46]. The closely related isoform PP2A-B55α has been shown to promote the timely reassembly of the nuclear envelope at mitotic exit [8]. Thus, the failure to reattach centrosomes to nuclei during mitotic exit in PP2A-Tws compromised embryos could be due to problems or a delay in nuclear envelope resealing.

Our results indicate that the proper regulation of the events of mitotic entry and exit by Polo and PP2A-Tws is crucial. This may be particularly true in the syncytial embryo due to the rapidity of the cycles, where one mitosis is almost immediately followed by another, and because of the obligatory cohesion between centrosomes and nuclei for their migration towards the cortex of the syncytium. Combining partial decreases in the activities of Polo and Tws strongly enhances the frequency of centrosome detachments observed (Figure 5). This suggests that when centrosomes fail to attach properly for too long between mitotic exit and the next mitotic entry, they become permanently detached from nuclei, leading to failures in mitotic divisions (Figure 5C).

The differences in timing between the detachments observed in *polo* and *tws* hypomorphic situations lead us to propose that the two enzymes act in parallel pathways, of which the disruption can lead to a failure in centrosome-nucleus cohesion. This is also supported by the prominent roles of Polo in regulating centrosome maturation and mitotic entry [2], and the specific requirements of PP2A-Tws/B55 at mitotic exit. However, we cannot exclude that Polo, Gwl and PP2A-Tws could function on a common substrate, or even in the same linear pathway, where the different players of the pathway could become more or less influential at different times of the cell cycle. In has been proposed that PP2A promotes full expression of Polo in larval neuroblasts and in S2 cells [47]. It has also been shown that depletion of Tws by RNAi leads to centrosome maturation defects in S2 cells [48], which could be explained by a reduction in Polo levels. However, we have repeatedly failed to detect a significant difference in Polo levels in embryos from *gwl^Scant^/+* or *tws/+* females, compared to wild-type controls by Western blotting (data not shown). Deeper genetic and molecular dissection of those pathways should lead to a clearer understanding of the regulation of centrosome and nuclear dynamics during mitotic entry and exit.

### A conserved pathway controls M-phase entry and exit

Our results add strong support to an emerging model for a pathway that controls entry into and exit from mitosis and meiosis in animal cells. It is increasingly clear that a form of PP2A associated with a B-type regulatory subunit plays a crucial and conserved role in competing with Cdk1. In Xenopus egg extract, PP2A-B55δ activity is high in interphase and low in M phase [9]. PP2A-B55δ must be down-regulated to allow mitotic entry, and conversely, it appears to promote mitotic exit both by inactivating Cdc25C and by dephosphorylating Cdk1 substrates [9], [46]. In human cells, depletion in B55α delays the events of mitotic exit, including nuclear envelope reassembly [8]. Already some years ago, mutations in Drosophila *tws* were found to lead to a mitotic arrest in larval neuroblasts [38], and extracts from *tws* mutants were shown to have a reduced ability to dephosphorylate Cdk substrates [49]. Mutations in *mts* resulted in an accumulation of nuclei in mitosis in the embryo [42]. The budding yeast now appears to be a particular case, as its strong reliance on the Cdc14 phosphatase to antagonize Cdk1 may reflect the need for insertion of the anaphase spindle through the bud neck prior to mitotic exit [50], a constraint that does not exist in animal cells. Nevertheless, additional phosphatases to PP2A, including PP1 are likely to play conserved roles in promoting mitotic and meiotic exit, and this remains to be dissected.

Our identification of PP2A genes as functional interactors of *polo* and *gwl* is the result of an unbiased genetic screen. We found that an elevation in Gwl function combined with a reduction in PP2A-Tws activity leads to a block in M phase, either in metaphase of meiosis I or in the early mitotic cycles. However, our positioning of Gwl as an antagonist of PP2A-Tws was facilitated by reports that appeared subsequent to our screen, proposing that the main role of Gwl in promoting M-phase was to lead to the inactivation of PP2A-B55δ in Xenopus egg extracts [43], [44]. Results consistent with this idea were also obtained in mammalian cells [26].

More recently, two seminal biochemical studies using Xenopus egg extracts showed that the antagonism of PP2A-B55δ by Gwl is mediated by α-endosulfine/Ensa and Arpp19, two small, related proteins which, when phosphorylated by Gwl at a conserved serine residue, become able to bind and inhibit PP2A-B55δ [51], [52]. By this mechanism, Gwl activation at mitotic entry leads to the inhibition of PP2A-B55δ, which results in an accumulation of the phosphorylated forms of Cdk1 substrates. Depletion of human Arpp19 also perturbs mitotic progression in Hela cells [51], suggesting a conserved role among vertebrates.

In an independent study, the group of David Glover has recently identified mutations in Drosophila *endosulfine* (*endos*) as potent suppressors of the embryonic lethality that occurs when *gwl^Scant^* (the gain-of-function allele) is combined with a reduction in *polo* function, in a maternal effect (see accompanying paper by Rangone et al [53]). *endos* is the single fly ortholog of Xenopus α-endosulfine and Arpp19. That the identification of *endos* by Rangone et al came from another unbiased genetic screen testifies of the specificity and conservation of the Gwl-Endos-PP2A pathway in animal cells. The authors went as far as showing that the critical phosphorylation site of Gwl in Endos is conserved between frogs and flies, and is critical for the function of Endos in antagonizing PP2A-Tws in cultured cells. These findings are consistent with a previous report showing that mutations in *endos* lead to a failure of oocytes to progress into meiosis until metaphase I [54]. Moreover, loss of Gwl specifically in the female germline also leads to meiotic failure, although in that case oocytes do reach metaphase I but exit the arrest aberrantly [23]. Although the meaning of those phenotypic differences is not yet understood, Gwl and Endos are both required for meiotic progression in Drosophila. Conversely, we show here that excessive Gwl activity relative to PP2A-Tws prevents exit from the metaphase I arrest, suggesting that the inhibition of PP2A-Tws by Gwl and Endos must be relieved to allow completion of meiosis. Moreover, Rangone et al show that the Endos pathway also regulates the mitotic cell cycle in the early embryo, in larval neuroblasts and in cultured cells [53].

Together, the systematic and unbiased identifications of mutations in PP2A-Tws subunit genes as enhancers (this paper), and of mutations in *endos* as suppressors [53] of *gwl^Scant^* provide strong evidence for a pathway connecting these genes to control M phase in flies. Our studies provide a convincing genetic and functional validation of the recent biochemical results from Xenopus extracts, and show that the Gwl-Endos-PP2A-Tws/B55 pathway is conserved and plays a key role in regulating both meiosis and mitosis in a living animal.

## Materials and Methods

### Fly husbandry, genetic screen, and fertility tests

Flies were kept at 25°C on standard food. The wild-type strain used was Oregon R. For the genetic screen, the DrosDel Core deletion kit [36](obtained from John Roote, Cambridge, UK) was used and uncovered approximately 60% of the genome with 200 deficiencies. For fertility tests, 3 to 5 well-fed, 1 to 4 days-old virgin females were given 3 to 5 Oregon R males per tube and allowed to mate for one day. Flies were then transferred on grape juice-containing agar and yeast. After one day, flies were removed (usually transferred to a new tube). Between 24 and 30 hours later, the percentage of hatched embryos was counted. At least 100 embryos were counted. *polo^11^*, *gwl^Scant^* and *gwl^Sr18^* alleles were previously published [23], [37]. *tws^P^* (*tws^J11C8^*) *and tws^aar-1^* were from David Glover. *mts^XE-2258^*, *Pp2A-29B^EP2332^*, *PP2A-B'^A131^*, and *wdb^07^* were from Bloomington stock center. *GFP-D-TACC* and *H2A-RFP* stocks were kindly provided by Pat O'Farrell and Jordan Raff. *UASp-GWL-MYC* and *UASp-GWL-KD-MYC* (K87R) expressed the long splice variant of Gwl, and were made in pPWM (Drosophila Genomics Resource Center). Transgenic flies were generated using the P-element-based method by BestGene Inc (Chino Hills, CA, USA). *UASp-GWL* flies were reported previously [23]. Overexpression in the early embryo was driven by maternal α-Tubulin Gal4, which was obtained from Adelaide Carpenter.

### Immunofluorescence and confocal microscopy

For immunofluorescence, embryos were collected on grape juice agar, and dechorionated and fixed as described [23]. Antibodies used for stainings are: α-Tubulin: YL1/2 (1:50; Serotec), γ-Tubulin: GTU-88 (1∶50; Sigma) and lamin B/Lamin Dm_0_ (1∶100; Developmental Studies Hybridoma Bank). Secondary antibodies were coupled to Alexa-488 (1∶200; Invitrogen) or Texas red (1∶200; Invitrogen). DNA was marked with DAPI. Images were acquired on a Laser scanning confocal microscope LSM 510 Meta (Zeiss), using a 100X oil objective.

### Time-lapse microscopy

Embryos from *WT* or *polo^11^/+* females and expressing *GFP-D-TACC* and *H2A-RFP* were dechorionated and imaged on a Swept-Field confocal microscope (Nikon Eclipse Ti), using a 100X oil objective.

### Chemical inhibition of Polo

Chemical treatments of embryos used a protocol modified from Sibon et al [29]. Embryos were dechorionated and incubated for 30 min in a 1∶1 mixture of Express Five Drosophila cell culture medium (Invitrogen) and heptane with 1 µM of BI2536 (from a DMSO stock solution) or DMSO alone.

## Supporting Information

Video S1 Time-lapse of an embryo from a *polo^11^/+* mother expressing GFP-D-TACC and H2A-RFP. Images were taken every 45 seconds on a Swept-field confocal microscope and with a 100X oil objective.
(AVI)

Video S2 Time-lapse of a WT embryo expressing GFP-D-TACC and H2A-RFP. Images were taken every 45 seconds on a Swept-field confocal microscope and with a 100X oil objective.
(AVI)
